# Effects of NO/cGMP inhibitors in a rat model of anaphylactoid shock

**DOI:** 10.1590/1414-431X20198853

**Published:** 2020-03-02

**Authors:** A.A.S. Albuquerque, L.G. Ferreira, M.T.M. Carvalho, V.K. Capellini, P.R.B. Evora, A.C. Celotto

**Affiliations:** 1 Departamento de Cirurgia e Anatomia, Faculdade de Medicina de Ribeirão Preto, Universidade de São Paulo, Ribeirão Preto, SP, Brasil; 2 Departamento de Biociências, Instituto de Saúde e Sociedade, Campus Baixada Santista, Universidade Federal de São Paulo, Santos, SP, Brasil; 3 Faculdade de Ciências da Saúde de Barretos Dr. Paulo Prata, Barretos, SP, Brasil

**Keywords:** Endothelial cells, Allergy, Rodent, Nitric oxide, Anaphylaxis

## Abstract

Anaphylactic shock can be defined as an acute syndrome, and it is the most severe clinical manifestation of allergic diseases. Anaphylactoid reactions are similar to anaphylactic events but differ in the pathophysiological mechanism. Nitric oxide (NO) inhibitors during anaphylaxis suggest that NO might decrease the signs and symptoms of anaphylaxis but exacerbate associated vasodilation. Therefore, blocking the effects of NO on vascular smooth muscle by inhibiting the guanylate cyclase (GC) would be a reasonable strategy. This study aimed to investigate the effects of NO/cGMP pathway inhibitors methylene blue (MB), N^ω^-nitro-L-arginine methyl ester hydrochloride (L-NAME), and indigo carmine (IC) in shock induced by compound 48/80 (C48/80) in rats. The effect was assessed by invasive blood pressure measurement. Shock was initiated by C48/80 intravenous bolus injection 5 min before (prophylactic) or after (treatment) the administration of the inhibitors MB (3 mg/kg), L-NAME (1 mg/kg), and IC (3 mg/kg). Of the groups that received drugs as prophylaxis for shock, only the IC group did not present the final systolic blood pressure (SBP) better than the C48/80 group. Regarding shock treatment with the drugs tested, all groups had the final SBP similar to the C48/80group. Altogether, our results suggested that inhibition of GC and NO synthase in NO production pathway was not sufficient to revert hypotension or significantly improve survival.

## Introduction

Anaphylactic shock can currently be defined as an acute syndrome, the most severe clinical manifestation of allergic diseases. Potentially fatal, it is a type I hypersensitivity reaction against specific antigens, leading to the formation of antibodies (1). The reaction requires sensitization to a particular substance which, on contact, produces immunoglobulin E (IgE) against the immunogen. When a new exposure occurs, the immune system responds immediately to mast cell degranulation (2–4).

Anaphylactoid reactions are like anaphylactic manifestations but differ in the pathophysiological mechanism. The pathogenesis of anaphylaxis typically involves IgE-dependent events, and the anaphylactoid responses include IgE-independent results that otherwise are clinically indistinguishable (1,5).

Anaphylactic and severe anaphylactoid reactions present the same clinical course and treatment (6,7). Mast cell degranulation releases inflammatory immune mediators that cause hypotension and shock.

It is known that nitric oxide (NO), an endogenous vasodilator, is associated with anaphylaxis. Moreover, experimental studies have shown that the inhibition of nitric oxide synthase (NOS) by L-arginine analogues reverses the hypotension caused by anaphylactic shock (8,9).

Histamine binding to H1 receptors during anaphylaxis also stimulates endothelial cells to convert the amino acid L-arginine into NO that activates guanylate cyclase (GC), leading to vasodilation and production of cyclic guanosine monophosphate (cGMP). Increased NO production decreases venous return, thus contributing to the vasodilation that occurs during anaphylaxis. NO inhibitors during anaphylaxis also promote bronchospasm, suggesting that NO might reduce the signs and symptoms of anaphylaxis but exacerbate associated vasodilation (10–12). Thus, blocking the effects of NO on vascular smooth muscle would inhibit GC, inhibiting vasodilation, being a reasonable strategy for anaphylaxis treatment.

Compound 48/80 (C48/80) is known to be a potent hypotensive agent and inducer of degranulation, responsible for releasing about 90% of histamine and other chemical mediators associated with symptoms of anaphylaxis and activation of mast cells (13,14). It has been used experimentally as a direct and convenient reagent to investigate the mechanisms of allergy and anaphylaxis, and, therefore, it has no clinical relevance, only experimental (15–17).

This study aimed to investigate the effects of NO/cGMP pathway inhibitors [methylene blue (MB), N^ω^-nitro-L-arginine methyl ester hydrochloride (L-NAME), and indigo carmine (IC)] in the treatment of induced shock by C48/80 in rats. The effects were assessed by observation of clinical signs, invasive blood pressure measurement, and the survival of animals in each experimental group (one of the main aims of the research).

## Material and Methods

### Ethics statement and animals

The animal procedures, as well as the experimental protocols of this study, were approved by the Ethics Committee on Animal Use (CEUA) of the University of São Paulo at Ribeirão Preto Medical School (protocol 190/210), according to the Ethical Principles in Animal Experimentation of the Brazilian College of Animal Experimentation (COBEA).

Male Wistar rats 70–90 days of age (300–350 g) were housed under standard laboratory conditions (12-h light/dark cycle at 22°C), with free access to food and water.

### Drugs

Urethane, C48/80, MB, L-NAME, and IC were from Sigma Chemical Company (USA) and all the drugs were prepared with distilled water.

### Experimental design and treatment protocol

The animals were anesthetized with urethane (2 mg/kg, *ip*) and maintained on spontaneous ventilation. After complete anesthesia, the inguinal region was cleaned and hair was removed. An incision was made and the femoral artery and vein were isolated and cannulated (24G × 0.75", Angiocath (Becton & Dickinson, Brazil). The femoral artery and vein were used for continuous measurement of systolic (SBP) and diastolic (DBP) blood pressure and drug administration, respectively. Blood pressure monitoring was performed using the MP System 100 A (Biopac System, Inc., USA) connected to a PC Gateway (Gateway, USA) with Windows XP operating system (Microsoft, USA) that can collect, analyze, store, and retrieve biophysical data. The vascular catheters were connected to pressure transducers, and these were connected to the continuous registration recorder MP System 100 A.

Shock was induced with C48/80 (3 mg/kg) by intravenous bolus injection, 5 min before (prophylactic) or after (treatment) the administration of the inhibitors [MB (3 mg/kg), L-NAME (1 mg/kg), IC (3 mg/kg)]. The doses for the drugs were chosen based on literature data (9,18–20).

Animals were randomly assigned into eleven groups (n=6 in each group): control, C48/80, MB, MB+C48/80, C48/80+MB, L-NAME, L-NAME+C48/80, C48/80+L-NAME, IC, IC+C48/80, and C48/80+IC. SBP and survival were analyzed over 60 min. SBP was examined every 10 min for 60 min or until death of the animal, whichever occurred sooner.

All animals that had a survival time of 60 min had blood samples collected from the femoral artery and placed in heparinized tubes (Eppendorf do Brasil, Brazil). Plasma was obtained by centrifugation at 2,500 *g* for 10 min at 4°C and immediately immersed in liquid nitrogen and freezer-stored (−70°C) to determine the nitrate/nitrite ratio. Plasma indirect dosages were performed by determining serum levels of nitrite and nitrate using the Sievers 280i NO Analyzer (Sievers, USA).

### Statistical analysis

Two-way ANOVA followed by Bonferroni *post*-test was used for statistical analysis of SBP. Moreover, for the analysis of the indirect measurements of the plasma NO, one-way ANOVA was used while for survival studies Kaplan-Meier method was chosen. The software used was GraphPad Prism version 5.0 (GraphPad Software Corporation, USA). The level of significance adopted was P<0.05.

## Results

As described in the literature (21), characteristics of anaphylactic shock hypotension were observed in the first minute after injection of the compound.

**Figure 1.**
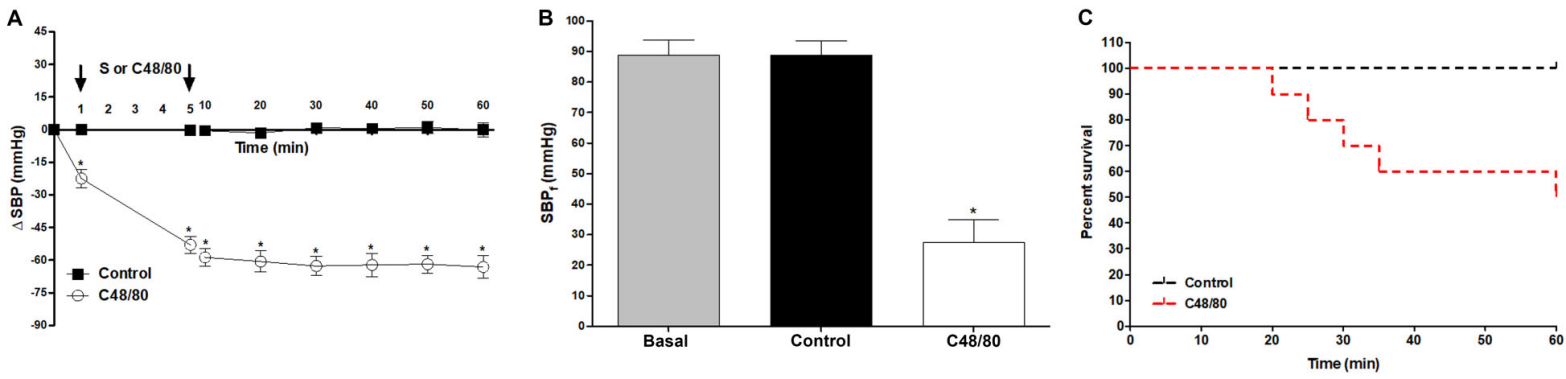
**A**, Systolic blood pressure (SBP), **B**, final systolic blood pressure (SBP_f_), and **C**, survival measurements of the control group and the group that received only C48/80. Data are reported as mean±SE. *P<0.001 C48/80 compared to control (**A**: two-way repeated-measures ANOVA and Bonferroni *post*-test; **B**: one-way ANOVA and Bonferroni *post*-test and **C**: Kaplan-Meier; n=6). S: saline.

C48/80 was efficient in shock induction because the rats presented hypotension, characteristic clinical signs such as cyanotic ears, paws, and tongue, and respiratory distress. The SBP and DBP decreased soon after C48/80 was administered and remained stable until the end of the experiment (Figure 1A and Table 1). Also, compared to the control group and basal measurement, it promoted a significant reduction in final systolic and diastolic blood pressures (Figure 1B and Table 1). Animal survival was 50% in the first hour after C48/80 infusion (Figure 1C).

**Table 1.** Initial and final systolic (SBP) and diastolic (DBP) blood pressures of the groups studied.

Groups	Initial	Final (60 min)
Control		
SBP	88.85±5.02	88.78±4.66
DBP	62.02±4.38	62.10±3.39
C48/80		
SBP	89.75±5.1	27.60±7.39
DBP	69.18±6.2	25.29±7.07
MB		
SBP	89.85±5.03	87.24±3.63
DBP	64.78±3.78	63.50±2.78
MB+C48/80		
SBP	92.18±5.48	37.73±7.30
DBP	69.15±4.06	30.98±5.47
C48/80+MB		
SBP	86.67±2.64	28.62±2.62
DBP	62.01±2.87	21.28±1.65
L-NAME		
SBP	83.59±5.23	110.27±4.07
DBP	55.11±4.21	88.60±3.39
L-NAME+C48/80		
SBP	102.68±6.27	43.63±8.08
DBP	76.15±7.32	37.14±6.68
C48/80+L-NAME		
SBP	89.12±7.07	29.67±5.89
DBP	64.60±5.25	24.63±5.31
IC		
SBP	97.11±1.88	94.25±5.34
DBP	68.67±2.25	67.14±4.68
IC+C48/80		
SBP	101.32±7.35	23.70±3.21
DBP	71.55±6.97	20.44±2.60
C48/80+IC		
SBP	103.98±6.51	30.24±5.16
DBP	76.14±6.06	23.99±3.74

Data are reported as mean±SE. MB: methylene blue; L-NAME: N^ω^-nitro-L-arginine methyl ester hydrochloride; IC: indigo carmine.

**Figure 2.**
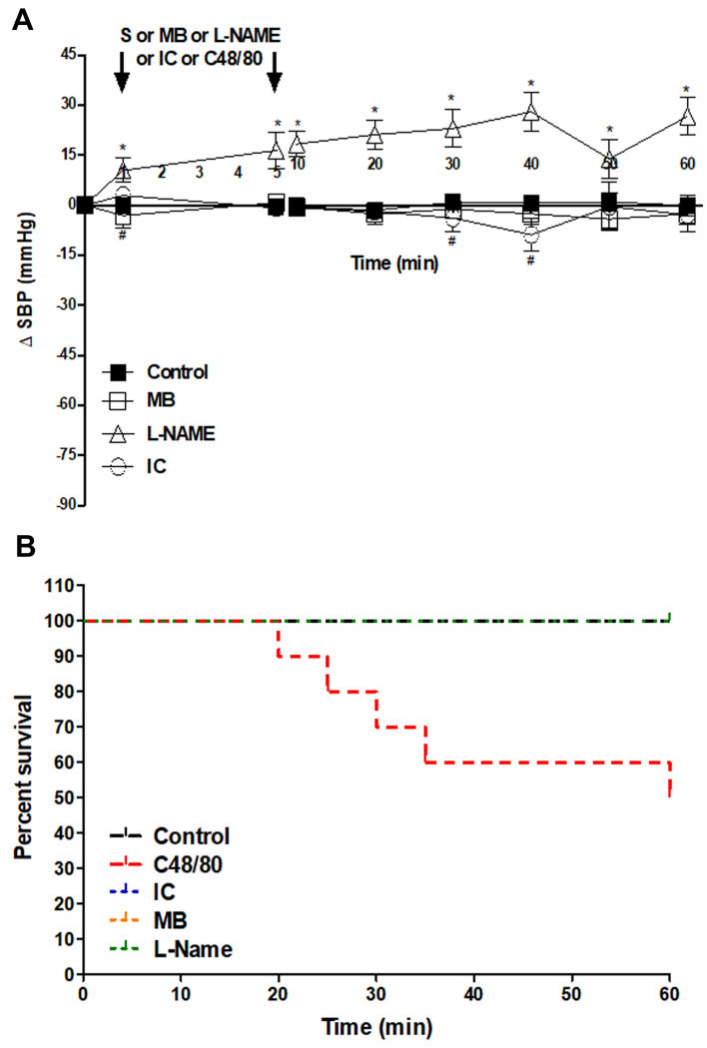
**A**, Systolic blood pressure (SBP) and **B**, survival evaluation of the control group and drug groups (MB, L-NAME, and IC). Data are reported as mean±SE. *P<0.001 L-NAME *vs* control; ^#^P<0.05 IC *vs* control. (**A**: two-way repeated-measures ANOVA and Bonferroni *post*-test; **B**: Kaplan-Meier*;* n=6). S: saline; MB: methylene blue; L-NAME: N^ω^-nitro-L-arginine methyl ester hydrochloride; IC: indigo carmine; S: saline.

Regarding survival, the group that received only MB or IC maintained the SBP (Figure 2A) and had 100% survival (Figure 2B), similar to the control group, during the 60 min that they were monitored. The L-NAME group remained with significantly higher SBP than the control group (Figure 2A), with 100% survival during the same time (Figure 2B).

### Methylene blue treatment

**Figure 3.**
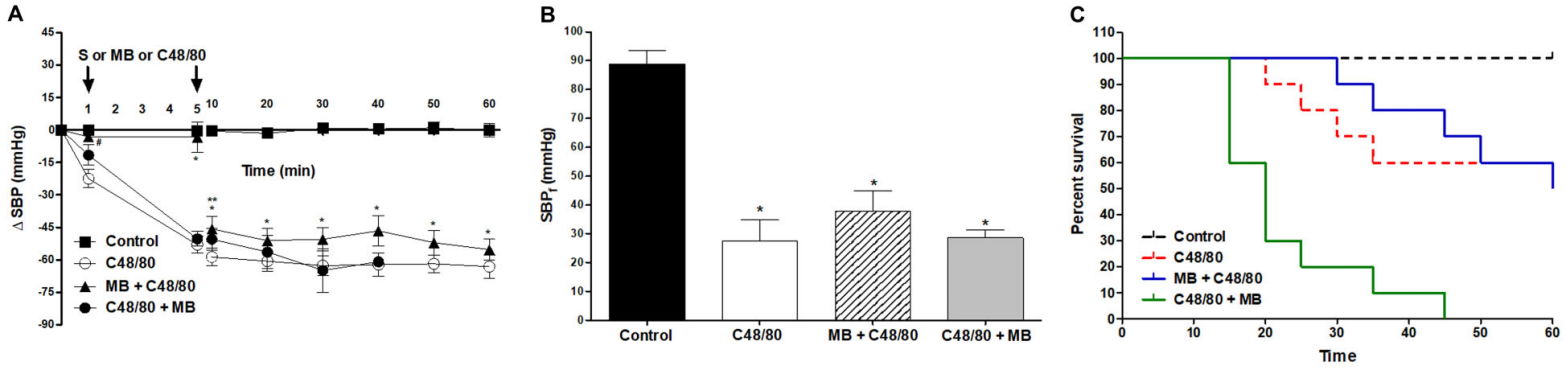
**A**, Systolic blood pressure (SBP), **B**, final systolic blood pressure (SBP_f_), and **C**, survival measurements of rats that received C48/80 and/or MB (Kaplan-Meier, n=6). Data are reported as mean±SE. **A**: *P<0.001 MB+C48/80 *vs* control; **P<0.001 MB+C48/80 *vs* C48/80; ^#^P<0.01 MB+C48/80 *vs* C48/80 (two-way repeated-measures ANOVA and Bonferroni *post*-test); **B**: *P<0.001 compared to control (one-way ANOVA and Bonferroni *post*-test). MB: methylene blue; S: saline.

The group that was given MB to prevent shock caused by C48/80 (MB+C48/80) presented better SBP (Figure 3A) and slightly higher final SBP (SBP_f_) (37±7 mmHg) compared to the group that received only the C48/80 (Figure 3B). However, in the MB treatment group (C48/80+MB), the SBP decreased after the compound infusion, and after the MB injection, a further decrease in SBP was observed (Figure 3A). Finally, the SBP_f_ was similar to the C48/80 group (28±2 mmHg) (Figure 3B). Survival was prolonged with MB pre-treatment, although it did not change the final survival. MB administration after C48/80 reduced survival time (60 to 45 min) (Figure 3C).

### L-NAME treatment

**Figure 4.**
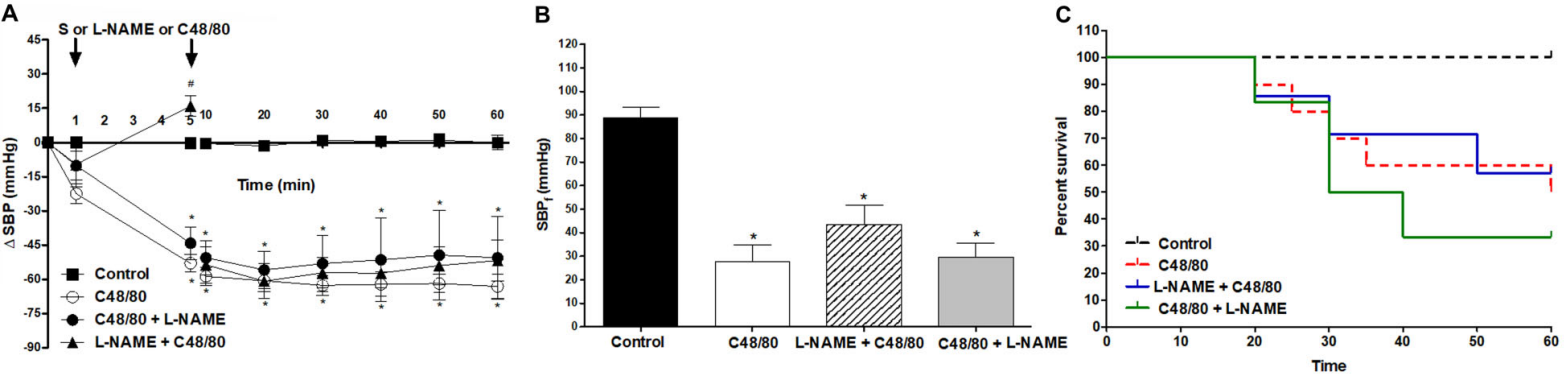
**A**, Systolic blood pressure (SBP), **B**, final systolic blood pressure (SBP_f_), and **C**, survival measurements of rats that received C48/80 and/or L-NAME (Kaplan-Meier, n=6). Data are reported as mean±SE. **A**: *P<0.001 C48/80, C48/80+L-NAME, L-NAME+C48/80 *vs* control; ^#^P<0.001 L-NAME+C48/80 *vs* C48/80 (two-way repeated-measures ANOVA and Bonferroni *post*-test); **B**: *P<0.001 compared to control (one-way ANOVA and Bonferroni *post*-test). L-NAME: N^ω^-nitro-L-arginine methyl ester hydrochloride; S: saline.

The SBP of the group in which L-NAME was used as prevention (L-NAME+C48/80) immediately increased when the drug was injected, but decreased significantly after induced shock, having a small improvement compared to the C48/80 group after 40 min (Figure 4A) (SBP_f_ 43±8 mmHg) (Figure 4B) and survival did not differ from C48/80 (Figure 4C). When animals received L-NAME as treatment for shock (C48/80+L-NAME), SBP showed a decrease when the compound was injected and no improvement with L-NAME. However, the treated group kept a better pressure than the compound group (Figure 4A). Moreover, the SBP_f_ was similar to the C48/80 group (29±5 mmHg) (Figure 4B). Survival was reduced with L-NAME after C48/80 (Figure 4C).

### IC treatment

**Figure 5.**
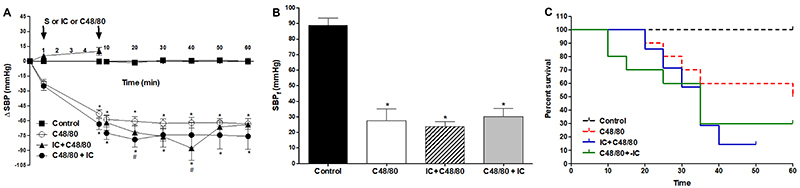
**A**, Systolic blood pressure (SBP), **B**, final systolic blood pressure (SBP_f_), and **C**, survival measurements (Kaplan-Meier; n=6) of rats that received C48/80 and/or IC. Data are reported as mean±SE. **A**: *P<0.001 C48/80, C48/80+IC, IC+C48/80 *vs* control; ^#^P<0.05 IC+C48/80, C48/80+IC *vs* C48/80 (two-way repeated-measures ANOVA and Bonferroni *post*-test); **B**: *P<0.001 compared to control (one-way ANOVA and Bonferroni *post*-test). IC: indigo carmine; S: saline.

IC administered for shock prevention (IC+C48/80) promoted a significant increase in SBP immediately after application (1 min), which remained until the 5th min when the C48/80 was injected. Then, the SBP decreased significantly and was maintained lower than the C48/80 group until the end of the experiment (Figure 5A) with a SBP_f_ of 23±3 mmHg (Figure 5B) and survival was a little worse than C48/80 group (Figure 5C).

As shock treatment (C48/80+IC), the dye did not alleviate the decrease in SBP, which remained lower than the C48/80 group (Figure 5A), ending the experiment with an SBP_f_ of 30±5 mmHg (Figure 5B) and survival of 30% in 60 min (Figure 5C).

### NO levels

**Figure 6.**
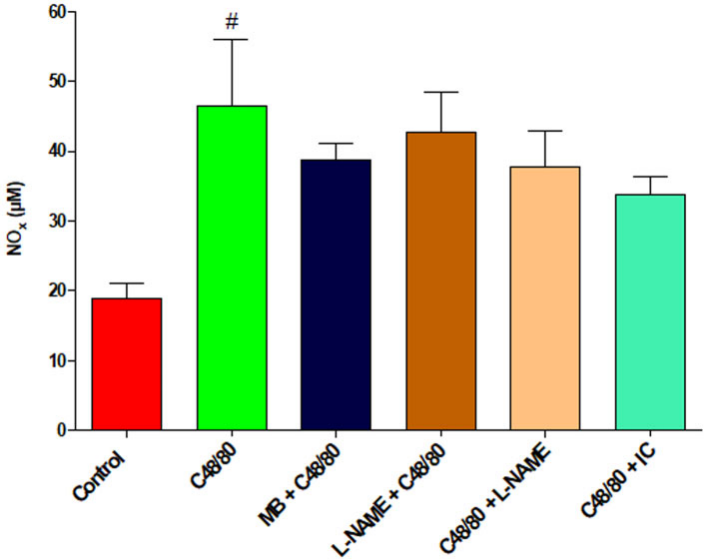
Plasma nitric oxide (NO) analysis of all groups. Data are reported as mean±SE.^#^P<0.01 compared to control (one-way ANOVA and Bonferroni *post*-test). MB: methylene blue; L-NAME: N^ω^-nitro-L-arginine methyl ester hydrochloride; IC: indigo carmine.

Analysis of the groups that received the drugs tested and that survived until the end of the study showed that plasma NO dosages between the groups were similar, with a statistically significant difference only between C48/80 group and the control group (Figure 6).

## Discussion

C48/80 has been used to produce experimental anaphylactic shock, because this compound is known to increase histamine release from plasma or tissue (22,23) and an additional nitric oxide release from endothelial cells (24). Our data showed that this compound was effective in inducing anaphylactic shock in rats since blood pressure decreased after C48/80 administration. In addition, the majority of the animals exposed to C48/80 presented cyanosis on ears, paws, and tongue, and respiratory distress. At the end of 60 min, all animals that received C48/80 showed a sudden drop in both systolic and diastolic pressure, practically equaling these pressures. The pulse pressure of almost zero justified the symptoms presented by the animals.

C48/80 acts by increasing the permeability of the lipid bilayer membrane of mast cells promoting disruption of the cell membrane, and mast cell degranulation by changing the free cytoplasmic calcium concentration, releasing mediators of anaphylaxis. Histamine, the most common mediator, connects to receptors on the endothelial cell membrane and triggers the synthesis of NO, resulting in vasorelaxation. However, some studies have shown that the C48/80 and other polybasic compounds are apparently capable of directly activating G proteins (25,26).

As observed in this work, other authors also demonstrated the efficiency of C48/80 in inducing anaphylactoid shock in mice (14,27,28), rats (15,29), guinea pigs (30), rabbits (18), and pigs (9). The ability of C48/80 to promote a direct release of nitric oxide from endothelial cells was confirmed by a significant increase in nitric oxide plasma levels. Unfortunately, none of the treatments employed was able to reduce nitric oxide levels, including L-NAME, probably because the release was high, considering the stimulus from histamine plus the direct effect on endothelium.

In some studies, the compound caused 100% mortality in animals (31), probably due to the potent hypotensive effect, mast cell degranulation, and nitric oxide release. It is also responsible for the release of about 90% of histamine and other chemical mediators as serotonin, associated with anaphylaxis symptoms (15,22).

One of the main aims of this study was to analyze animal survival with substances used as treatment and prevention of anaphylactoid shock. To avoid or minimize the hypotension caused by C48/80, we decided to use the NO pathway inhibitors, because it is the main final mediator of the vasodilation pathway.

MB was chosen as a non-selective inhibitor of GC. Therefore, it was expected that when the action of this enzyme was blocked, cGMP would not increase, thus inhibiting vasodilatation. However, the group that received the prophylactic MB did not show a significant stabilization of blood pressure. Furthermore, treated animals showed worse hypotension and increased mortality, compared to the C48/80 group. These findings corroborate the results of Takano et al. (27) who used MB as prophylaxis to shock induced by the compound in mice. On the other hand, it differs from the results reported by Buzato et al. (18) as they observed a significant recovery of blood pressure in rabbits receiving MB as a treatment to shock.

The prophylactic use of MB has been shown to be effective in improving survival of rabbits after C48/80-induced shock (18). However, in this experimental model, prophylactic MB did not improve survival from C48/80 shock.

The survival evaluation results in an experiment with rabbits were more faithful to the effects observed in humans who had anaphylactic shock and were treated with MB (32) than with the experimental mouse model. These results showed that there may have been a blockage of GC when MB was used as prevention, resulting in enough decrease in vasodilation to increase survival by 70% compared to the treated group.

The clinical use of MB for the treatment of anaphylactic shock shows excellent results in more than 20 cases. However, anaphylactic shock is a medical emergency and there is no evidence that MB was the primary choice, and thus it is challenging to design a study that would be in line with ethical principles. In these studies, MB therapy was only started after treatment with conventional drugs like vasopressors, corticosteroids, antihistamines, and fluids was carried out and did not work. In some cases, hypotension reversal occurred immediately after the first dose, but in others, a second dose or even a continuous infusion of MB was required.

In this study, L-NAME was also not effective in recovering hypotension caused by shock when used as both prevention and treatment. SBP remained close to the values of the C48/80 group, showing a slight increase after 20 min. However, the use of L-NAME caused an improvement in the shock group, possibly because the endothelial nitric oxide synthase (eNOS) was blocked by the inhibition of NO production, with a consequent SBP increase. Differing from these findings, L-NAME attenuated anaphylactic hypotension in mice (33), reduced vasodilation induced by histamine in the mesenteric region and upper limbs in cats, and reduced vasodilation of venules in pigs and cerebral arteries in monkeys (34). The isolated L-NAME infusion can reduce cardiac output, promoting bronchoconstriction and increasing anaphylaxis mortality. The use of NOS inhibitors in the treatment of anaphylactic shock is still questionable.

The treated rats had a higher mortality than the C48/80 group and the group that received the substance as prophylaxis improved survival around 25%. Cauwels et al. (35) observed a similar result when using mice sensitized with bovine serum albumin. The vasodilation can be explained by its dependence on eNOS, which maintains hypotension and causes circulatory shock and mortality (27).

IC, besides acting by blocking the endothelium-dependent vasodilation, also affects peripheral alpha constrictors (36,37). IC when used as a blue dye for urologic and gynecologic procedures to identify ureteral patency occasionally causes side effects that include hypertension. This effect is associated with a vasoconstriction effect (38). However, few cases of severe hypotension after IC injection have been reported in the literature (39,40).

IC, contrary to expectations, was not effective in improving the survival of animals either as prophylaxis or as treatment. Considering the ambiguous effect of IC on blood pressure, at this time, it is difficult to understand the mechanism involved in the present results.

In the adopted animal model of anaphylactoid shock, severe hypotension associated with impairment of tissue perfusion and high mortality was observed. This condition was probably mediated by increased plasma NO. However, the blockade in the NO/cGMP pathway with MB, L-NAME, or IC as treatment did not improve the low blood pressure or mortality caused by C48/80. On the other hand, survival time increased when the substances were applied prophylactically. It is possible that in this anaphylactic model the shock was too severe, hampering the prevention only by NO/cGMP pathway blockade.
